# Neuropeptide Y as Alternative Pharmacotherapy for Antidepressant-Resistant Social Fear

**DOI:** 10.3390/ijms21218220

**Published:** 2020-11-03

**Authors:** Johannes Kornhuber, Iulia Zoicas

**Affiliations:** Department of Psychiatry and Psychotherapy, Friedrich-Alexander University Erlangen-Nürnberg (FAU), 91054 Erlangen, Germany; Johannes.Kornhuber@uk-erlangen.de

**Keywords:** social fear, social anxiety, social investigation, fear extinction, fear expression, SSRI, paroxetine, tricyclic antidepressants, amitriptyline, NPY

## Abstract

In many social anxiety disorder (SAD) patients, the efficacy of antidepressant therapy is unsatisfactory. Here, we investigated whether mice deficient for the lysosomal glycoprotein acid sphingomyelinase (ASM−/−) represent an appropriate tool to study antidepressant-resistant social fear. We also investigated whether neuropeptide Y (NPY) reduces this antidepressant-resistant social fear in ASM−/− mice, given that NPY reduced social fear in a mouse model of SAD, namely social fear conditioning (SFC). We show that neither chronic paroxetine nor chronic amitriptyline administration via drinking water were successful in reducing SFC-induced social fear in ASM−/− mice, while the same treatment reduced social fear in ASM+/− mice and completely reversed social fear in ASM+/+ mice. This indicates that the antidepressants paroxetine and amitriptyline reduce social fear via the ASM-ceramide system and that ASM−/− mice represent an appropriate tool to study antidepressant-resistant social fear. The intracerebroventricular administration of NPY, on the other hand, reduced social fear in ASM−/− mice, suggesting that NPY might represent an alternative pharmacotherapy for antidepressant-resistant social fear. These results suggest that medication strategies aimed at increasing brain NPY concentrations might improve symptoms of social fear in SAD patients who fail to respond to antidepressant treatments.

## 1. Introduction

With a lifetime prevalence of approximately 12% [1], social anxiety disorder (SAD) is the second most common anxiety disorder after specific phobia and is characterized by the persistent fear and avoidance of social situations. This avoidant behavior is essential for the maintenance of SAD, as it prevents the reversal of fear in social situations. The best treatment outcomes are currently obtained through cognitive behavioral therapy, especially with exposure therapy, which leads to a gradual decline in the fear response because of repeated exposure to the feared situation, a process termed fear extinction [2]. This psychotherapy is often combined with a rather unspecific pharmacotherapy consisting of antidepressants, benzodiazepines, beta-blockers, anticonvulsants, or neuroleptics, with selective serotonin reuptake inhibitors (SSRIs) providing the best response rates [3]. Many SAD patients, however, fail to respond to these treatment options, achieve only partial remission of symptoms, and/or show a high rate of relapse after treatment discontinuation [4,5], thus highlighting the necessity for more efficient therapies.

Neuropeptides are viable research candidates due to their role in social behaviors and stress-related behaviors. Neuropeptide Y (NPY), a 36-amino acid peptide, is the most abundant and widely distributed neuropeptide in the mammalian brain. NPY is expressed in brain areas involved in social behavior and the fear circuitry, such as the amygdala, hippocampus, septum, periaqueductal gray, locus coeruleus, cerebral cortex, basal ganglia, hypothalamus, and thalamus [6,7]. Its biological effects are mediated through five subtypes of G*_i_*-protein-coupled receptors termed Y1, Y2, Y4, Y5, and Y6 [8]. NPY and its receptors regulate important biological and pathophysiological functions, including blood pressure, energy homeostasis, neuroendocrine secretions, neuronal excitability, and neuroplasticity [9,10,11,12]. NPY also causes a variety of behavioral effects when administered into the brain in rodents. For example, NPY stimulates food intake when administered into the hypothalamic paraventricular nucleus [13], promotes social interaction when administered into the dorsolateral septum [14] and basolateral amygdala [15], and has anxiolytic and antidepressant-like effects when administered intracerebroventricularly (i.c.v.) [16,17]. NPY also affects different aspects of fear-related behaviors, as shown in fear conditioning studies in rodents. For example, i.c.v.-administered NPY impairs the acquisition and consolidation of cued and contextual fear [18,19,20], impairs the expression of fear memories [21], and facilitates the extinction of cued and contextual fear [20,21]. These prosocial, anxiolytic, and fear-reducing properties of NPY suggest its potential benefit in disorders associated with social anxiety and fear. Accordingly, we could show that i.c.v.-administered NPY reduces the expression of social fear in an animal model of SAD, namely social fear conditioning (SFC) [22]. SFC is an animal model that mimics the major behavioral symptoms of SAD, i.e., reduced social investigation and avoidance of conspecifics as indicative of social fear [23,24]. Importantly, both acute treatment with the benzodiazepine diazepam and chronic treatment with the SSRI paroxetine reversed social fear in socially fear-conditioned (SFC^+^) mice [23], providing a predictive validity to the SFC model. 

Recently, it was shown that the effects of antidepressant drugs like amitriptyline and fluoxetine are mediated by the acid sphingomyelinase (ASM)-ceramide system [25]. ASM is a lysosomal glycoprotein that catalyzes the hydrolysis of sphingomyelin to ceramide and phosphorylcholine at acidic pH [26]. Tricyclic antidepressants like desipramine, imipramine, and amitriptyline and SSRIs like fluoxetine, paroxetine, and sertraline induce the proteolytic degradation of ASM and, thereby, functionally inhibit its activity [27,28]. It is well-known that chronic corticosterone treatment reduces hippocampal neurogenesis, neuronal maturation, and neuronal survival and induces depressive-like symptoms in rats and mice [25,29]. These corticosterone-induced alterations could be normalized by chronic amitriptyline and fluoxetine treatments both in wild-type mice and in mice overexpressing ASM (ASMtg). This same treatment in ASM-deficient mice (ASM−/−), however, failed to exert antidepressant-like effects and to normalize the corticosterone-induced alterations, indicating an antidepressant-resistant phenotype in ASM−/− mice [25]. 

Considering that many SAD patients fail to respond to antidepressant therapies, we investigated whether antidepressants also fail to reverse symptoms of social fear in ASM−/− mice and whether these mice might represent an appropriate tool for studying antidepressant-resistant social fear. As neither paroxetine nor amitriptyline treatment were successful in reducing SFC-induced social fear in ASM−/− mice, we investigated whether NPY might reduce social fear and thereby represent an alternative therapeutic approach for antidepressant-resistant social fear. 

## 2. Results

### 2.1. Chronic Paroxetine Treatment (10 mg/kg/Day for 14 Days) Reduces Social Fear in ASM+/+ and ASM+/− Mice but Not in ASM−/− Mice

Given that chronic paroxetine administration (10 mg/kg/day via the drinking water for 14 days) reversed SFC-induced social fear in CD1 mice [23], we determined whether this same treatment affects social fear in ASM−/− mice. For this, we studied conditioned (SFC^+^) and unconditioned (SFC^−^) ASM−/−, ASM+/−, and ASM+/+ mice that were administered paroxetine (10 mg/kg/day via the drinking water for 14 days), starting one day after SFC to prevent possible treatment-induced alterations in fear memory consolidation. During SFC on day 1, SFC^+^ mice received mild electric foot shocks each time they investigated an unknown conspecific, whereas SFC^−^ mice investigated an unknown conspecific without receiving foot shocks. On day 16, during social fear extinction, we assessed the time that the SFC^+^ and SFC^−^ mice spent investigating three empty cages (i.e., non-social investigation) and six unknown conspecifics (i.e., social investigation) as a read-out of non-social and social fear, respectively. 

**Figure 1:**
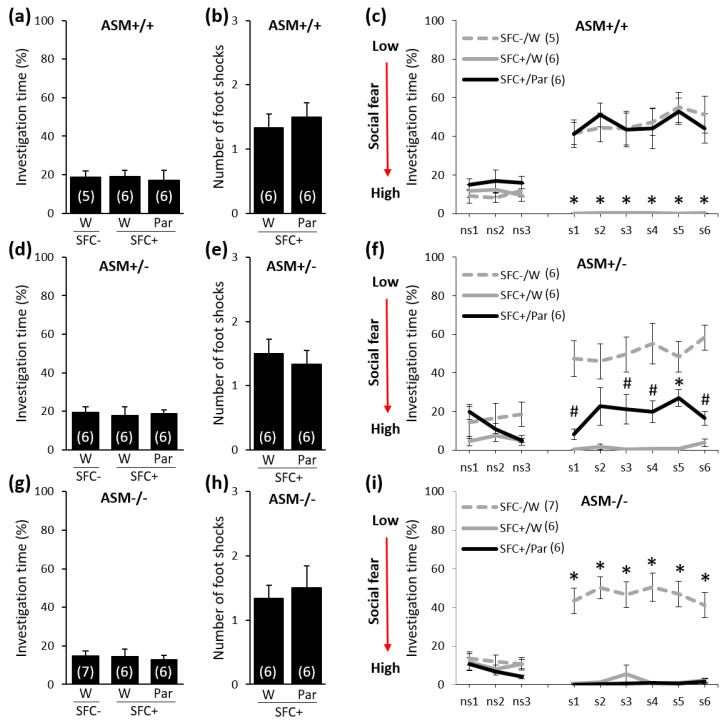
Chronic paroxetine treatment (10 mg/kg/day for 14 days) reduces social fear in ASM+/+ and ASM+/− mice but not in ASM−/− mice. (**a**,**d**,**g**) Preconditioning investigation of the non-social stimulus (empty cage) shown by unconditioned (SFC^−^) and conditioned (SFC^+^) mice during SFC on day 1. (**b**,**e**,**h**) Number of foot shocks received by SFC^+^ mice during SFC. (**c**,**f**,**i**) Investigation of the non-social (ns1–ns3) and social (cages with mice; s1–s6) stimuli during social fear extinction on day 16. Paroxetine (Par) was administered over 14 days in the drinking water (W) starting one day after SFC. Data represent mean ± SEM, and numbers in parentheses indicate group sizes. *p* < 0.05 * versus all groups; # versus SFC^−^/W.

During SFC on day 1, all mice spent a similar amount of time investigating the non-social stimulus (empty cage), which indicates similar preconditioning non-social anxiety between the groups before treatment (Figure 1a: F(2,14) = 0.049; *p* = 0.952, Figure 1d: F(2,15) = 0.062; *p* = 0.940, and Figure 1g: F(2,16) = 0.135; *p* = 0.875). Furthermore, SFC^+^ mice received a similar number of foot shocks during SFC, indicating similar levels of distress during SFC (Figure 1b: T(10) = −0.542; *p* = 0.599, Figure 1e: T(10) = 0.542; *p* = 0.599, and Figure 1h: T(10) = −0.415; *p* = 0.687). On day 16, during social fear extinction, all SFC^+^ and SFC^−^ mice showed similar investigations of the non-social stimuli (three empty cages; ns1–ns3), which indicates that SFC did not induce an unspecific non-social fear (Figure 1c,f,i). However, all water-drinking SFC^+^ mice spent less time investigating the social stimuli (six unknown conspecifics, s1–s6) compared with respective water-drinking SFC^−^ mice, indicating increased social fear. Paroxetine, on the other hand, completely reversed social fear in ASM+/+ mice (Figure 1c; stimulus x group effect F(16,112) = 7.752; *p* < 0.001) and reduced social fear in ASM+/− mice (Figure 1f; stimulus x group effect F(16,120) = 6.137; *p* < 0.001) but did not reduce the social fear in ASM−/− mice (Figure 1i; stimulus x group effect F(16,128) = 11.966; *p* < 0.001). This indicates that paroxetine alters the expression of social fear in a genotype-dependent manner and that ASM−/− mice do not respond to this paroxetine treatment.

### 2.2. Chronic Paroxetine (20 mg/kg/Day for 28 Days) or Amitriptyline (180 mg/L for 28 Days) Treatment Reduces Social Fear in ASM+/+ and ASM+/− Mice but Not in ASM−/− Mice

To determine whether a higher dose of paroxetine or longer treatment duration might be necessary to reverse social fear in ASM−/− mice, we administered paroxetine (20 mg/kg/day) via the drinking water over 28 days, starting one day after SFC. We also determined whether the tricyclic antidepressant amitriptyline (180 mg/L, for 28 days) could reverse social fear. Social fear was assessed on day 30 during social fear extinction.

**Figure 2:**
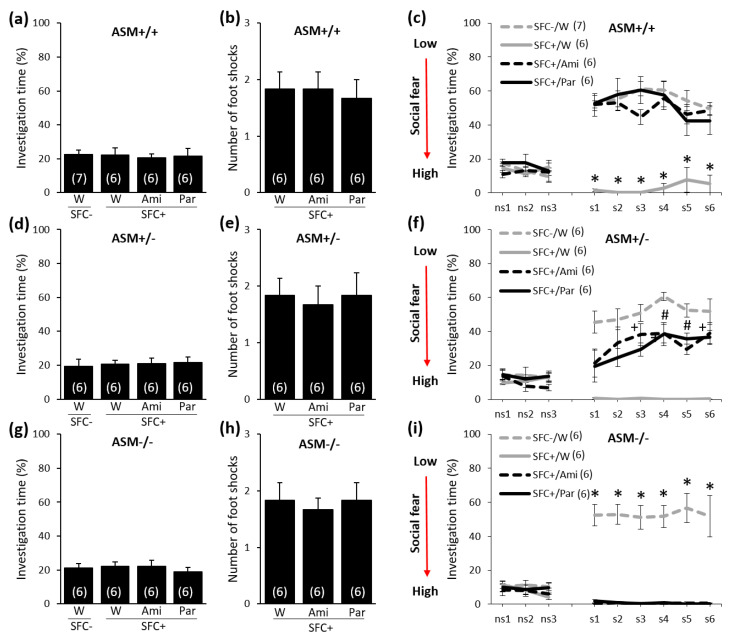
Chronic paroxetine (20 mg/kg/day for 28 days) or amitriptyline (180 mg/L for 28 days) treatment reduces social fear in ASM+/+ and ASM+/− mice but not in ASM−/− mice. (**a**,**d**,**g**) Preconditioning investigation of the non-social stimulus (empty cage) shown by unconditioned (SFC^−^) and conditioned (SFC^+^) mice during SFC on day 1. (**b**,**e**,**h**) Number of foot shocks received by SFC^+^ mice during SFC. (**c**,**f**,**i**) Investigation of the non-social (ns1–ns3) and social (cages with mice, s1–s6) stimuli during social fear extinction on day 30. Paroxetine (Par) or amitriptyline (Ami) were administered over 28 days in the drinking water (W) starting one day after SFC. Data represent means ± SEM, and numbers in parentheses indicate group sizes. *p* < 0.05 * versus all groups, # versus SFC^−^/W and SFC^+^/W, and + versus SFC^+^/W.

During SFC on day 1, all mice showed similar preconditioning non-social anxiety before treatment (Figure 2a: F(3,21) = 0.055; *p* = 0.982, Figure 2d: F(3,20) = 0.086; *p* = 0.967, and Figure 2g: F(3,20) = 0.292; *p* = 0.831), and all SFC^+^ mice received a similar number of foot shocks (Figure 2b: F(2,15) = 0.093; *p* = 0.912, Figure 2e: F(2,15) = 0.076; *p* = 0.927, and Figure 2h: F(2,15) = 0.119; *p* = 0.889). On day 30, during social fear extinction, all SFC^+^ and SFC^−^ mice showed similar non-social investigation (Figure 2c,f,i), which indicates that SFC did not induce unspecific non-social fear. While all water-drinking SFC^+^ mice showed high levels of social fear, both paroxetine and amitriptyline reversed social fear in ASM+/+ mice (Figure 2c; stimulus x group effect F(24,168) = 10.484; *p* < 0.001) and reduced social fear in ASM+/− mice (Figure 2f; stimulus x group effect F(24,160) = 6.574; *p* < 0.001) but did not reduce social fear in ASM−/− mice (Figure 2i; stimulus x group effect F(24,160) = 15.431; *p* < 0.001). This indicates that neither the SSRI paroxetine nor the tricyclic antidepressant amitriptyline could reduce social fear in ASM−/− mice. 

### 2.3. Acute NPY Treatment Reduces Social Fear in ASM+/+, ASM+/−, and ASM−/− Mice

Given that i.c.v.-administered NPY (1 nmol/2 µL) reduced SFC-induced social fear in CD1 mice [22], we determined whether NPY could also reduce the antidepressant-resistant social fear in ASM−/− mice. For this, SFC^+^ and SFC^−^ ASM−/−, ASM+/−, and ASM+/+ mice were infused i.c.v. either with NPY or with a vehicle solution (Veh) 10 min before social fear extinction on day 16.

**Figure 3:**
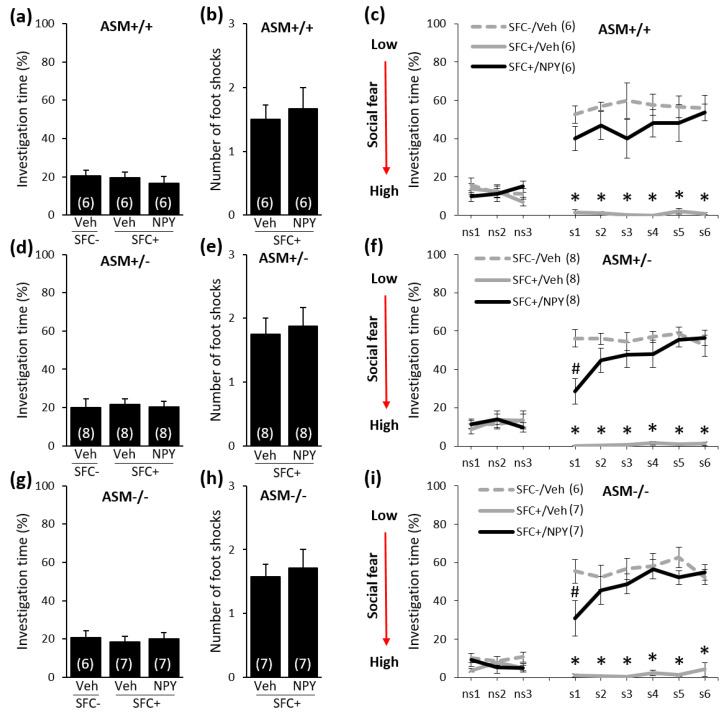
Acute neuropeptide Y (NPY) treatment reduces social fear in ASM+/+, ASM+/−, and ASM−/− mice. (**a**,**d**,**g**) Preconditioning investigation of the non-social stimulus (empty cage) shown by unconditioned (SFC^−^) and conditioned (SFC^+^) mice during SFC on day 1. (**b**,**e**,**h**) Number of foot shocks received by SFC^+^ mice during SFC. (**c**,**f**,**i**) Investigation of the non-social (ns1–ns3) and social (cages with mice, s1–s6) stimuli during social fear extinction on day 16. NPY (1 nmol/2 µL) or a vehicle solution (Veh; 2 µL) was administered intracerebroventricularly (i.c.v.) 10 min before social fear extinction. Data represent means ± SEM, and numbers in parentheses indicate group sizes. *p* < 0.05 * versus all groups; # versus SFC^−^/Veh.

During SFC on day 1, all mice showed similar preconditioning non-social anxiety before treatment (Figure 3a: F(2,15) = 0.418; *p* = 0.666, Figure 3d: F(2,21) = 0.060; *p* = 0.942, and Figure 3g: F(2,17) = 0.106; *p* = 0.900) and all SFC^+^ mice received a similar number of foot shocks (Figure 3b: T(10) = −0.415; *p* = 0.687, Figure 3e: T(14) = −0.323; *p* = 0.751, and Figure 3h: T(12) = −0.408; *p* = 0.690). On day 16, during social fear extinction, all SFC^+^ and SFC^−^ mice showed similar non-social investigation (Figure 3c,f,i), which indicates that SFC did not induce unspecific non-social fear. While all vehicle-treated SFC^+^ mice showed high levels of social fear, NPY reduced social fear in ASM+/+ mice (Figure 3c; stimulus x group effect F(16,120) = 13.627; *p* < 0.001), ASM+/− mice (Figure 3f; stimulus x group effect F(16,168) = 20.777; *p* < 0.001), and in ASM−/− mice (Figure 3i; stimulus x group effect F(16,136) = 15.284; *p* < 0.001). This indicates that NPY can reduce symptoms of social fear in ASM−/− mice and might represent an alternative therapeutic approach for antidepressant-resistant social fear.

## 3. Discussion

Our study shows for the first time that paroxetine and amitriptyline fail to reduce symptoms of social fear in ASM−/− mice. NPY, on the other hand, reduces social fear in ASM−/− mice, indicating that NPY might represent an alternative pharmacotherapy for antidepressant-resistant social fear.

We were able to replicate our previous findings showing that chronic paroxetine treatment for two weeks reverses SFC-induced social fear in male CD1 mice [23] and then extended these findings by showing that this treatment also reverses social fear in wild-type ASM+/+ mice. In ASM−/− mice lacking the lysosomal glycoprotein ASM, however, two weeks of paroxetine treatment did not reverse social fear, while, in ASM+/− mice, that showed a lower ASM activity compared with ASM+/+ mice [25], paroxetine partly reversed social fear, suggesting that ASM mediates the effects of paroxetine on social fear. Interestingly, prolonged paroxetine treatment with higher doses (i.e., for four weeks with 20 mg/kg/day instead of two weeks with 10 mg/kg/day), while ineffective in ASM−/− mice, seemed to be more effective in ASM+/− mice. While two weeks of paroxetine treatment significantly reduced social fear only during the investigation of the second and fifth social stimulus (Figure 1f), four weeks of paroxetine treatment reduced social fear starting from the first social stimulus (Figure 2f). Four weeks of amitriptyline treatment induced comparable effects to those observed after four weeks of paroxetine treatment, suggesting that the SSRI and the tricyclic antidepressant treatment used in this study shows similar efficacy against symptoms of social fear in the SFC model of SAD.

An antidepressant-resistant phenotype has been previously described in ASM−/− mice [25]. As such, chronic amitriptyline and fluoxetine treatment for four weeks failed to exert anxiolytic and antidepressant-like effects in unstressed and in corticosterone-stressed ASM−/− mice, although these effects were observed both in wild-type mice and in mice overexpressing ASM (ASMtg). This antidepressant treatment also failed to alter hippocampal neurogenesis, neuronal maturation, and neuronal survival in unstressed ASM−/− mice and to normalize the corticosterone-induced decrease in hippocampal neurogenesis, neuronal maturation, and neuronal survival. The same treatment in wild-type and ASMtg mice, however, increased the hippocampal neurogenesis, neuronal maturation, and neuronal survival in unstressed mice and normalized the corticosterone-induced alterations, suggesting that ASM−/− mice do not respond to this antidepressant treatment [25]. As in our study, amitriptyline and fluoxetine demonstrated similar efficacy. Considering that neither tricyclic antidepressants nor SSRIs were successful in alleviating symptoms of social fear (this study), depression, or anxiety in ASM−/− mice [25], these mice seem to represent a viable tool not only to investigate the molecular mechanisms involved in the resistance to antidepressants but, also, to test novel drugs with different ways of action. 

Given that i.c.v.-administered NPY reduced SFC-induced social fear in CD1 mice [22], we investigated whether NPY might also reduce the antidepressant-resistant social fear in ASM−/− mice. We demonstrated that acute NPY treatment did not only reduce social fear in ASM+/+ and ASM+/− mice but, also, in ASM−/− mice. This supports previous findings showing that i.c.v.-administered NPY reduced the expression and facilitated the extinction of cued and contextual fear [20,21]. These results also demonstrate that NPY reduces social fear independent of the ASM-ceramide system and might therefore represent an alternative pharmacotherapy for antidepressant-resistant social fear.

Although experiments were performed in both sexes and we did not see any sex differences within the groups, the relatively low number of mice used in this study makes a clear statement on a possible sex-dependent effect of antidepressants and/or NPY on social fear impossible at this stage. Similarly, it is not clear whether sex differences are to be expected in the role of ASM in mediating the effects of SSRIs and tricyclic antidepressants on social fear.

Although the mechanisms underlying the effects of NPY on social fear are not known yet, these might include modulatory effects of NPY on corticosterone secretion and on cardiovascular function. As such, i.c.v.-administered NPY increased the plasma corticosterone concentrations [30] and increased corticosterone concentrations via the intraperitoneal or intrabasolateral amygdala administration of glucocorticoid receptor agonists that were shown to facilitate fear extinction [31]. On the other hand, i.c.v.-administered NPY is known to blunt elevations in blood pressure and heart rate after exposure to the resident-intruder paradigm, an established model of social stress [32], and to blunt fear-induced tachycardia in a cued fear conditioning paradigm [33]. By reducing cardiovascular function in response to stressful stimuli, NPY might enable SFC^+^ mice to approach social stimuli more quickly and might lead, therefore, to a faster extinction of social fear. 

Interestingly, all control SFC^+^ mice (i.e., water-drinking and vehicle-treated) used in this study failed to show extinction of social fear, i.e., a gradual decrease in social fear as a result of repeated exposure to social stimuli. Gradual social fear extinction has been previously described in untreated and vehicle-treated CD1 mice [22,23,34,35,36,37,38,39] and is similar to the outcome during exposure therapy in SAD patients [2]. This difference in social fear extinction might arise from potential genetic-related strain differences. Whereas CD1 mice are outbred mice that reflect the genetic diversity in the human population better, the ASM-deficient mice and wild-type littermates used in this study are inbred C57BL/6 mice. Differences in anxiety-related behavior [40,41], depressive-like behavior [42,43], and social behavior [44,45] were previously reported in these strains. As such, C57BL/6 mice are more anxious, showing higher levels of depressive-like behavior and a more affiliative type of social behavior. Although no differences in non-aggressive social behavior were observed between C57BL/6 and CD1 mice, CD1 mice engaged more in aggressive behaviors, resulting in a higher total time spent in social interactions [44,45]. These high levels of aggression might motivate SFC^+^ CD1 mice to approach the social stimuli faster and might therefore lead to a faster extinction of social fear. Alternatively, the more anxious and depressive-like phenotypes of C57BL/6 mice might hinder SFC^+^ ASM+/+ mice from approaching the social stimuli. However, ASM−/− mice were less anxious [25,46] and showed lower levels of depressive-like behavior compared with ASM+/+ mice [25]. Given that this reduced anxiety did not seem to facilitate social approach, i.e., SFC^+^ ASM+/+, ASM+/−, and ASM−/− mice showed similarly impaired extinction of social fear, it is more likely that the differences in aggressive-like behavior between C57BL/6 and CD1 mice affect the extinction rate. These strain differences in social behavior were not only observed in SFC^+^ mice but, also, in SFC^−^ mice, i.e., SFC^−^ ASM+/+, ASM+/−, and ASM−/− mice showed lower levels of social investigation during social fear extinction compared with SFC^−^ CD1 mice. SFC^−^ CD1 mice usually spend around 80% of the time investigating the social stimuli [22,23,34,35,36,37,38,39] and show high levels of aggressive behavior, whereas SFC^−^ C57BL/6 mice used in this study spent around 60% of the time investigating the social stimuli and showed no aggressive behavior. 

Taken together, we show that neither chronic paroxetine treatment nor chronic amitriptyline treatment were successful in reversing social fear in ASM−/− mice, suggesting that ASM mediates the effects of SSRIs and tricyclic antidepressants on social fear. We also show that acute NPY treatment reduces social fear in ASM−/− mice and might therefore represent an alternative pharmacotherapy for antidepressant-resistant social fear. These results suggest that medication strategies aimed at increasing brain NPY concentrations might improve symptoms of social fear in SAD patients who fail to respond to antidepressant therapies. Such strategies might include the administration of brain-penetrating NPY receptor agonists or of inhibitors of NPY-cleaving peptidases.

## 4. Materials and Methods

### 4.1. Animals

Homozygous (ASM−/−; *Smpd1*−/−) and heterozygous (ASM+/−; *Smpd1*+/−) ASM-deficient mice and wild-type littermates (ASM+/+; 8–12 weeks old) were studied in sex-balanced designs. Mice were individually housed for one week before experiments started and remained single-housed throughout experiments. Mice were held under standard laboratory conditions (12:12 light:dark cycle, lights on at 07:00 h, 22 °C, 60% humidity, food and water ad libitum). Experiments were performed during the light phase, between 09:00 and 14:00, in accordance with the Guide for the Care and Use of Laboratory Animals of the Government of Unterfranken (project identification code 55.2-2532.1-27/11 approved on 7 September 2015 and project identification code 55.2 2532-2-314 approved on 13 December 2016) and the guidelines of the NIH. All efforts were made to minimize animal suffering and to reduce the number of animals used.

### 4.2. Social Fear Conditioning (SFC) Paradigm

To induce social fear, mice were conditioned during SFC on day 1, and social investigation was assessed during social fear extinction on day 16 or on day 30 as a read-out of social fear. 

SFC. SFC was performed with a computerized fear conditioning system (TSE System GmbH, Bad Homburg, Germany), as previously described [22,29,34,35,36,37,38,39] (see [24] for a schematic representation of the SFC paradigm). Mice were placed in the conditioning chamber (45 × 22 × 40 cm), and after a 30-sec habituation period, an empty wire mesh cage (7 × 7 × 6 cm) was placed as a non-social stimulus near one of the short walls. After 3 min, the non-social stimulus was replaced by an identical cage containing an unfamiliar sex- and age-matched ASM+/− mouse. Unconditioned mice (SFC^−^) were allowed to investigate this social stimulus for 3 min, whereas conditioned mice (SFC^+^) were given a 1-sec mild electric foot shock (0.7 mA) each time they investigated, i.e., made direct contact with the social stimulus. Mice received between one and three foot shocks with a variable inter-shock interval depending on when direct social contact was made. The number of foot shocks was assessed as a measure of distress and of social fear learning. Mice were returned to their home cage when no further social contact was made for 2 min (average duration of SFC approximately 10 min). The time the mice spent investigating the non-social stimulus as a preconditioning measure of non-social anxiety was analyzed.

### 4.3. Social Fear Extinction

On day 16 or on day 30, mice were exposed in their home cage to three non-social stimuli, i.e., empty cages identical to the cage used during SFC, to assess non-social investigation as a parameter of non-social fear. Mice were then exposed to six unfamiliar social stimuli, i.e., mice enclosed in wire mesh cages, to assess social investigation as a parameter of social fear. Each stimulus was placed near a short wall of the home cage and presented for 3 min, with a 3-min inter-exposure interval. The test was recorded and analyzed using JWatcher (Version 1.0, Macquarie University, Sydney, Australia and UCLA, Los Angeles, CA, USA). Non-social investigation was defined as the direct sniffing of the empty cage, whereas social investigation was defined as direct sniffing of the cage and/or of the social stimulus inside of the cage.

### 4.4. Antidepressant Treatment

Paroxetine (paroxetine hydrochloride; Sigma-Aldrich, Darmstadt, Germany) was administered via the drinking water at a dose of 10 or 20 mg/kg/day, as previously described [23]. Amitriptyline (amitriptyline hydrochloride; Sigma-Aldrich, Darmstadt, Germany) was administered via the drinking water at a dose of 180 mg/L, as previously described [25,47]. Treatment was started one day after SFC to avoid possible treatment-induced alterations in fear memory consolidation. 

### 4.5. Stereotaxic Cannula Implantation

Implantation of the guide cannula (21 G, 8-mm length; Injecta GmbH, Klingenthal, Germany) for i.c.v. infusions was performed under ketamine-xylazine anesthesia (intraperitoneal injection of 120 mg/kg Ketavet and 16 mg/kg Rompun, respectively), as previously described [22,34,36,48], 2 mm above the right lateral ventricle (from Bregma: +0.2 mm, lateral: +1.0 mm, depth: +1.4 mm). After surgery, mice were handled for five days before the experiment started.

### 4.6. Intracerebral Infusions

Mice received i.c.v. infusions of either vehicle (Veh; distilled H_2_O; 2 µL) or porcine NPY (1 nmol/2 µL; PeptaNova, Sandhausen, Germany) via an infusion cannula (23 G, 10-mm length) inserted into the guide cannula and connected via polyethylene tubing to a Hamilton syringe. The infusion system remained in place for 30 sec following infusion to allow diffusion of the solution.

The correct infusion site was verified by infusing ink post-mortem and verifying the coloration of the ventricular system; accordingly, all guide cannulas were implanted correctly. NPY dose and timing of administration was selected based on previous studies [19,22,48].

### 4.7. Statistical Analysis

For statistical analysis, SPSS (Version 24, SPSS Inc., Chicago, IL, USA) was used. Data were analyzed using the Student’s *t*-test, one- or two-way ANOVA for repeated measures followed by a Bonferroni’s post-hoc analysis whenever appropriate. Statistical significance was set at *p* < 0.05.
